# Impact of sports interventions on aggressive behavior among adolescents: a systematic review and meta-analysis

**DOI:** 10.3389/fpsyg.2025.1697324

**Published:** 2025-11-06

**Authors:** Hongliang Wang, Shiwei Chen, Wenling Gou, Xue Han

**Affiliations:** 1 School of Physical Education and Health, Yancheng Normal University, Yancheng, China; 2 School of Health Sciences, Universiti Sains Malaysia, Seberang Perai, Kelantan, Malaysia; 3 Faculty of Physical and Sports Sciences and Techniques, University of Montpellier, Montpellier, France; 4 Rehabilitation Center, Hebei Institute of Sports Science, Shijiazhuang, China; 5 Key Laboratory of Training Load Diagnosis and Regulation for Elite Athletes, General Administration of Sport of China, Shijiazhuang, China; 6 Hebei Key Laboratory of Digital Physical Fitness Monitoring and Health Promotion, Shijiazhuang, China

**Keywords:** adolescent, aggression, hostility, anger, sports interventions

## Abstract

**Introduction:**

Aggression in adolescents adversely affects developmental and mental health outcomes. Sports participation has been proposed as a potential way to reduce aggression by improving self-control and social skills; however, the evidence remains inconsistent. Therefore, this study systematically evaluated the effects of sports interventions on aggression, hostility, and anger in adolescents.

**Methods:**

A systematic literature search was performed in PubMed, Embase, the Web of Science, the Cochrane Library, and EBSCOhost-SPORTDiscus up to 15 July 2025. Meta-analysis was conducted to calculate standardized mean differences (SMDs) for aggression outcomes using a random-effects model. Subgroup analyses and sensitivity tests were performed to explore the sources of heterogeneity.

**Results:**

A total of 11 studies with 1,811 participants were included. For aggression, no significant overall effect of sports interventions was found (I^2^ = 86%, Hedges’ g = 0.46, 95% CI − 0.24 to 1.16). Subgroup analysis by sport type also showed no significant effects. For hostility, a significant reduction was observed (I^2^ = 0.0%, Hedges’ g = 0.29, 95% CI 0.13 to 0.45). For anger, no significant overall effect was found (I^2^ = 77.2%, Hedges’ g = 0.32, 95% CI − 0.19 to 0.84). Subgroup analysis showed a significant effect for non-contact sports (I^2^ = 9.5%, Hedges’ g = 0.52, 95% CI 0.18 to 0.86), but no significant effect was observed for contact sports.

**Conclusion:**

Sports interventions may reduce hostility but have no significant impact on aggression and anger in adolescents. Non-contact sports showed a significant effect in reducing anger. Meanwhile, no significant effects were found for aggression or anger in contact sports, suggesting that while sports interventions could help mitigate hostility, their effectiveness in addressing aggression and anger requires further investigation. Further longitudinal studies are needed to confirm long-term effects and clarify the psychological mechanisms, such as self-control and social skills, through which sports may influence aggression-related outcomes.

## Introduction

Aggression, defined as a behavior intended to cause harm or injury to others, represents a major developmental challenge during adolescence (Zirpoli, 2008; Fauzi et al., 2023). Such behaviors hinder psychological growth, social adaptation, and academic achievement and are associated with an increased risk of later mental health problems, such as anxiety and depression (Undheim and Sund, 2010; Gini and Pozzoli, 2013; Gini et al., 2014). Epidemiological studies indicate that approximately half of middle school students exhibit elevated levels of aggressive behavior, with the prevalence rising throughout adolescence (Hamza et al., 2019). Considering these adverse consequences, researchers have sought effective strategies to mitigate aggressive tendencies, with one promising approach being participation in sports and structured physical activities.

Sports participation may serve as a constructive channel for arousal and frustration, offering adolescents a socially acceptable way to express and regulate emotions (Fite and Vitulano, 2011; Karin et al., 2010). It also provides repeated practice in self-control, cooperation, and adherence to rules, developing core social–emotional skills such as emotional regulation and teamwork, which are linked to lower levels of aggression, hostility, and anger (Holt et al., 2020; Lubans et al., 2012; Pesce et al., 2021). Recent research further supports this view, showing that sports participation may buffer the effects of early adverse experiences on aggression by strengthening emotion regulation and promoting prosocial functioning (Hoeven et al., 2024; Janković et al., 2024a). Through these processes, sports are believed to reduce outwardly visible problematic behaviors such as aggression and rule-breaking (Poitras et al., 2016).

However, empirical findings remain inconsistent. Several studies have reported that athletes tend to exhibit higher levels of aggression, and even among students, greater participation in physical activity has sometimes been associated with increased aggression scores (Sønderlund et al., 2014; Kreager, 2007; Méndez et al., 2019). In contrast, other studies have found that regular participation in sports is associated with lower aggression and improved emotional control (Karin et al., 2010; Kim, 2016). These mixed results suggest that the relationship between sports and aggression may depend on contextual factors, such as the type of sport and individual life-course patterns of participation (Janković et al., 2024b). These conflicting results make it difficult to determine whether sports can reliably reduce aggressive behavior in adolescents.

Based on these inconsistencies, this study aims to systematically review and conduct a meta-analysis on the effects of sports participation on adolescent aggression. Specifically, it examines three primary outcomes—aggression, hostility, and anger—and explores potential moderators such as sport type, intervention duration, and frequency.

By quantitatively integrating available evidence, this study seeks to clarify the relationship between sports participation and aggressive behavior and to provide practical insights for schools, communities, and policymakers seeking effective behavioral prevention strategies.

## Methods

This review was performed in accordance with the PRISMA statement (Page et al., 2021), and the protocol was prospectively registered in the PROSPERO database (CRD420251126130).

### Search strategy and selection

A systematic literature search was performed in PubMed, Embase, the Web of Science, the Cochrane Library, and EBSCOhost-SPORTDiscus, covering all records from database inception to 15 July 2025. These databases were chosen for their complementary strengths in medicine, biomedicine, multidisciplinary science, evidence-based interventions, and sports research. To minimize the risk of missing relevant studies, the reference lists of the related systematic reviews and included articles were also manually screened. The search strategy combined terms related to sports interventions (e.g., exercise, sport*, physical activity, physical education, fitness training, and motor activity), adolescents (e.g., adolescent, adolescents, teen, teens, youth, young people, high school students, middle school students, and juvenile), and aggression-related outcomes (e.g., aggression, aggressive behavior, aggressive behavior, hostility, hostile behavior, hostile behavior, bullying, and fighting). Boolean operators (“AND” and “OR”) were used to combine these terms, and appropriate subject headings (e.g., MeSH in PubMed) were applied when available; full search strategies are provided in Supplementary File 1. In addition, the reference lists of pertinent systematic reviews and meta-analyses were manually checked to identify any additional eligible studies.

After removing duplicate records, two reviewers (H.W. and S.C.) independently screened titles and abstracts for relevance. The full texts of studies deemed potentially eligible were subsequently retrieved and evaluated against the predefined inclusion criteria. Any discrepancies were resolved through discussion with W.G., and unresolved issues were adjudicated by X.H.

### Eligibility criteria

Studies were included if they met the following criteria: Participants were adolescents participating in school-based or community-based settings. The intervention involved sports- or exercise-based programs (e.g., structured sports activities, physical training, and physical education programs) explicitly designed or implemented as part of the study. The comparison group was a control group receiving no sports intervention, usual activities, or alternative non-sports interventions. Outcomes were quantitative assessments of aggression or related constructs, specifically aggression scores, hostility, and anger, measured using validated instruments or behavior observation scales. Eligible study designs included randomized controlled trials or quasi-experimental studies with both an intervention group and a control group. Studies were required to report sufficient statistical information to calculate effect sizes, specifically providing means and standard deviations (or data convertible to these) for aggression outcomes in both groups at baseline and post-intervention.

Eligible study designs were limited to randomized controlled trials or quasi-experimental studies to ensure a high level of methodological quality and reduce the influence of confounding variables. Focusing on high-quality experimental evidence was intended to provide the most reliable estimates of intervention effects, while acknowledging that this decision may limit the breadth of the included evidence.

Studies were excluded if they targeted populations outside the adolescent age range, did not include a control/comparison group, used interventions not involving sports or structured physical activity, did not report aggression-related outcomes, or lacked sufficient data to compute effect sizes.

### Data extraction

Data from each included study were extracted independently by H.W., S.C., and W.G. using a standardized form. The following information was recorded: author, year of publication, country, study design, participant characteristics (e.g., sample size, mean age, and gender distribution), intervention details (type, frequency, duration, and setting), outcome measures (aggression scores, hostility, and anger), and moderator variables (e.g., sport type, intervention duration, and frequency).

For the primary analysis, mean values and standard deviations (SDs) of aggression-related outcomes at baseline and post-intervention were extracted for both the intervention and control groups. When change scores (post–pre) were reported, these were used directly; otherwise, change scores were calculated when sufficient data were available. If SDs were not reported, they were derived from standard errors, confidence intervals (CIs), t-values, or *p*-values using established formulas. When data were presented only in graphical form, values were extracted from figures using a digital tool by two independent reviewers and cross-checked to minimize potential error.

If necessary, corresponding authors were contacted by email up to two times within a three-week period to obtain missing or clarifying data. Consistency between data extractors was verified through repeated cross-checking, and all unresolved discrepancies were adjudicated by X.H.

### Risk of bias

The risk of bias for all included studies was assessed using the Risk of bias In Non-randomized Studies of Interventions (ROBINS-I) tool (Sterne et al., 2016). The ROBINS-I tool covers seven domains: bias due to confounding, bias in the selection of participants, bias in the classification of interventions, bias due to deviations from intended interventions, bias due to missing data, bias in the measurement of outcomes, and bias in the selection of the reported result. Each domain was rated as “low risk,” “moderate risk,” “serious risk,” or “critical risk” of bias, following the guidance of the tool. The assessments were performed independently by H.W. and S.C., with disagreements resolved through discussion with W.G. and all unresolved disagreements adjudicated by X.H. The results of the risk-of-bias assessment were not only used to guide sensitivity analyses but were also considered when interpreting the pooled results and evaluating the overall strength of the evidence.

### Publication bias and sensitivity analysis

Funnel plot asymmetry and Egger’s regression test were used to assess potential publication bias, with a *p*-value of > 0.05 indicating no statistically significant bias (Peters et al., 2008). These analyses were conducted only for aggression, as at least 10 studies were available. For hostility (five studies) and anger (six studies), neither Egger’s test nor the trim and fill procedure was applied because, with such a small number of studies, these methods have very low statistical power and may yield misleading results (Sterne et al., 2011).

**Figure 1:**
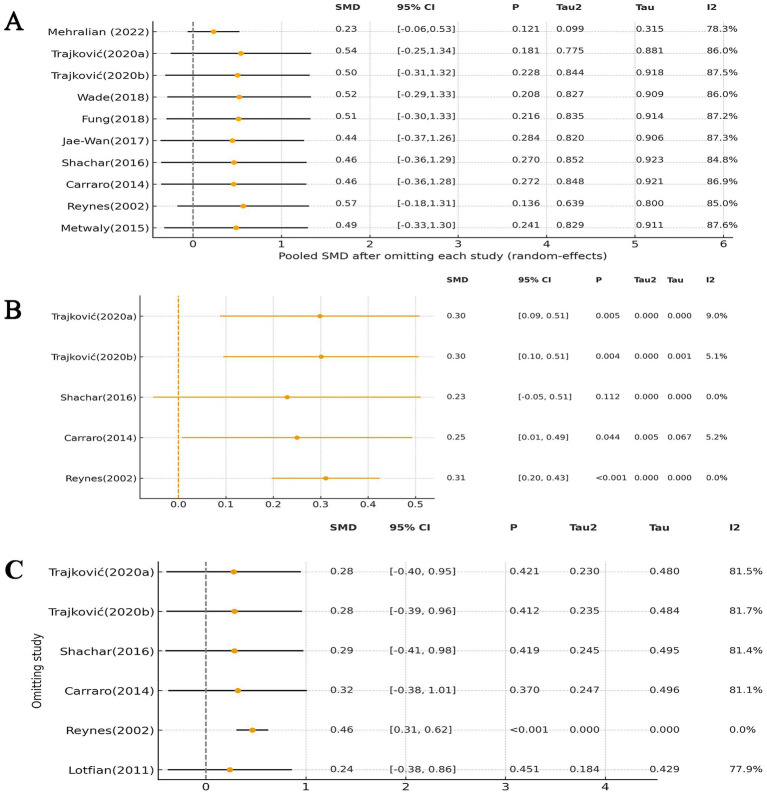
Leave-one-out sensitivity analyses: **(A)** aggression outcome; **(B)** hostility outcome; **(C)** anger outcome.

Sensitivity analyses were performed using a leave-one-out approach, in which each study was sequentially removed to examine its influence on the pooled effect estimates and between-study heterogeneity (Marušić et al., 2020). The stability of the results was evaluated by comparing the pooled standardized mean differences (SMDs) and heterogeneity statistics (I^2^ and Tau^2^) across iterations. The results of the sensitivity analyses are presented in the main text (Figure 1).

### Statistical analysis

Meta-analyses were conducted to synthesize the effects of sports interventions on aggression-related outcomes by comparing post-intervention values between the intervention and control groups. Effect sizes were expressed as standardized mean differences (SMDs; Hedges’g) with 95% confidence intervals (CIs). A random effects model was used to account for expected heterogeneity across the studies (Borenstein et al., 2009). Between-study variance was estimated using restricted maximum likelihood (REML), and the Hartung–Knapp adjustment was applied to improve the accuracy of random effects CIs (Partlett and Riley, 2017). Positive SMD values indicated lower levels of aggression-related outcomes (e.g., reduced aggression, hostility, or anger) in the intervention group compared to the control group.

Statistical heterogeneity was assessed using the I^2^ statistic, with values of 25, 50, and 75% considered indicative of low, moderate, and high heterogeneity, respectively (Chen et al., 2025). When substantial heterogeneity (I^2^ > 50%) was detected, subgroup analyses were considered to explore potential sources. A subgroup analysis was conducted based on sport type (contact vs. non-contact). Although additional subgroup analyses (e.g., based on intervention duration, frequency, or intervention type) and meta-regression were planned, they could not be performed due to the limited number of studies and inconsistent reporting of moderator variables. All analyses were performed using R (version 4.4.0; R Foundation for Statistical Computing, Vienna, Austria) with the meta and metafor packages. Two-tailed *p*-values of < 0.05 were considered statistically significant.

## Results

### Study selection and basic characteristics

**Figure 2:**
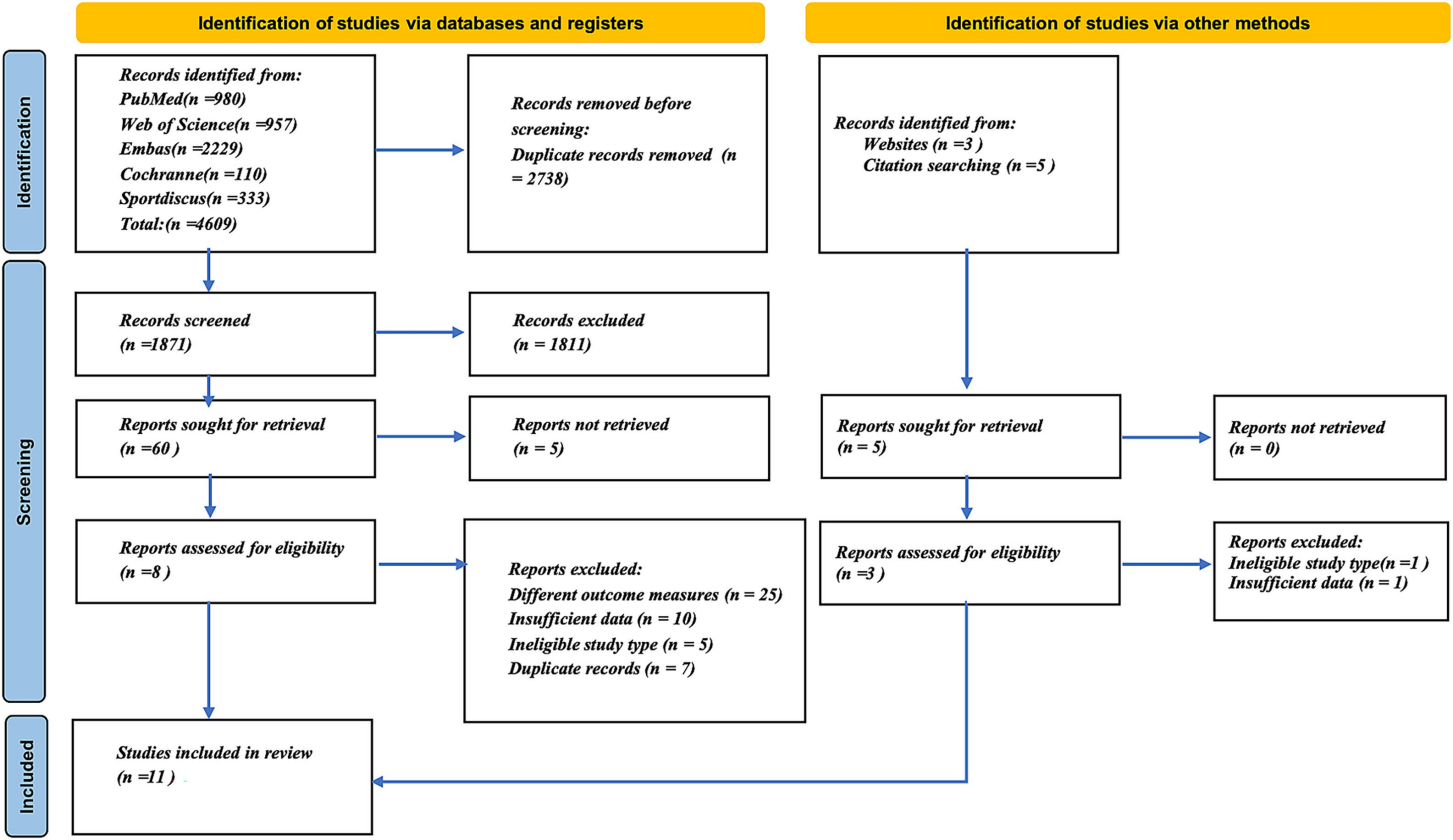
PRISMA 2020 flow diagram showing the process of study identification, screening, eligibility assessment, and inclusion.

As of 28 July 2025, a total of 4,609 records were retrieved through database searches. The initial screening yielded 60 potentially eligible studies. Following the full-text review, a total of 11 studies were included in the meta-analysis (Mehralian et al., 2022; Trajković et al., 2020a; Trajković et al., 2020b; Wade et al., 2018; Fung and Lee, 2018; Park et al., 2017; Shachar et al., 2016; Carraro et al., 2014; Reynes and Lorant, 2002; Metwaly, 2015; Lotfian et al., 2011). A detailed flowchart of the study selection process is shown in Figure 2.

These 11 studies collectively involved 1811 participants. All included studies compared post-intervention outcomes between exercise intervention groups and non-intervention control groups. Outcomes consisted of quantitative assessments of aggression and related constructs, specifically aggression, hostility, and anger. The duration of intervention periods varied across the studies. Key characteristics of the included studies are summarized in Table 1.

**Table 1:** Characteristics of the included literature.

Author	Country	N (total sample)	Measure	Duration frequency	% Female	Age (years)	Intervention
Mehralian et al. (2022)	Iran	30	Aggression	10 training sessions, each lasting 1 h	N/A	7–10	Yoga
Trajković et al. (2020a)	Serbia	107	Aggression Hostility Anger	8 months, 2 × 45-min sessions/week, ≥1-day apart	E:30% C:37%	E:15.5 ± 0.7C:15.7 ± 0.6	Small-sided volleyball sessions
Trajković et al. (2020b)	Serbia	105	Aggression Hostility Anger	64 after-school sessions over 8 months (45 min, 2/week, ≥1-day apart)	E:26% C:31%	E:15.7 ± 0.6C:15.8 ± 0.5	Recreational soccer sessions
Wade et al. (2018)	Australia	289	Aggression	8 months	0%	12.7 ± 0.5	A school-based, multicomponent physical activity
Fung and Lee (2018)	Hong Kong	139	Aggression	10–90-min weekly sessions	E:21% C:32%	E:8.63 ± 1.06C:8.57 ± 1.11	Martial arts
Park et al. (2017)	Korea	48	Aggression	8 weeks	50%	E:12.03 ± 0.83C:12.29 ± 0.65	Physical education class
Shachar et al. (2016)	Israel	649	Aggression Hostility Anger	24 weeks, 5 h/week	24%	N/A	Extracurricular sports activities
Carraro et al. (2014)	Italy	210	Aggression Hostility Anger	4 weeks, 8 lessons, 2 times a week	42%	13.27 ± 0.48	Play-fighting activities
Reynes and Lorant (2002)	France	55	Aggression Hostility Anger	1 year, 2 sessions per week	N/A	8	Judo practice
Metwaly (2015)	Egypt	24	Aggression	1 h per day, 2 times a week for ten weeks	N/A	E:5 ± 1.9C:5 ± 1.2	Hydro gymnastics training
Lotfian et al. (2011)	Iran	155	Anger	5.8 h/week	N/A	E:13.73 ± 2.22C:16.28 ± 0.95	Swimming

E = Experimental group; C = Control group.

### Risk of Bias and publication Bias

**Figure 3:**
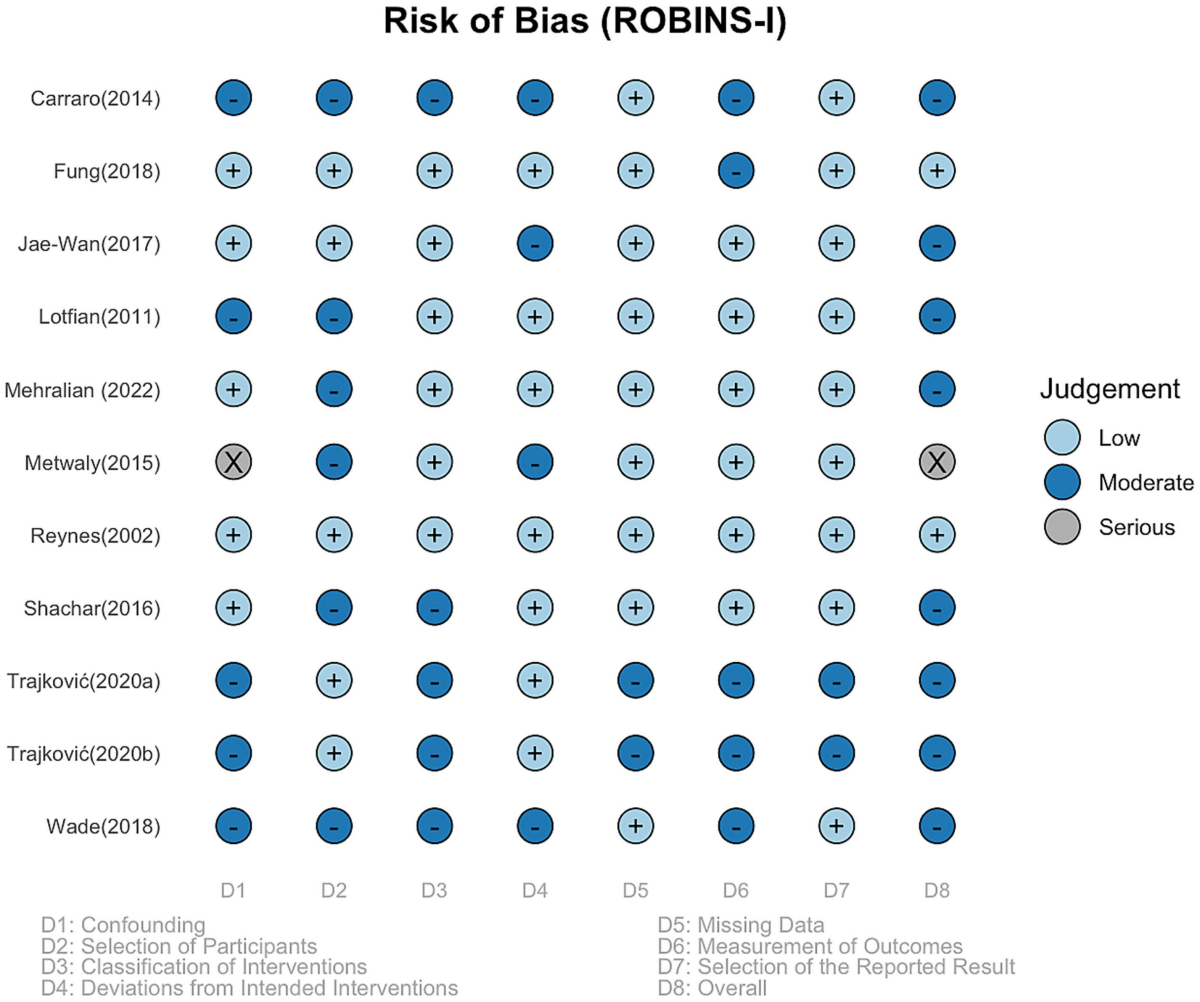
Risk of bias assessment of the included studies using the ROBINS-I tool.

Overall, the risk of bias assessment using the ROBINS-I tool indicated that most studies were at low to moderate risk of bias (Figure 3). The majority of studies showed a low risk of bias in the domains of confounding, participant selection, and missing data. Moderate risk ratings were primarily observed in the domains of intervention classification, deviations from intended interventions, and outcome measurement, reflecting limitations in the reporting of intervention fidelity and blinding procedures. Only one study (Metwaly, 2015) was rated as having a serious risk of bias due to concerns across multiple domains. Taken together, these findings suggest that while the overall methodological quality was acceptable, potential sources of bias remain and should be considered when interpreting the pooled results.

**Figure 4:**
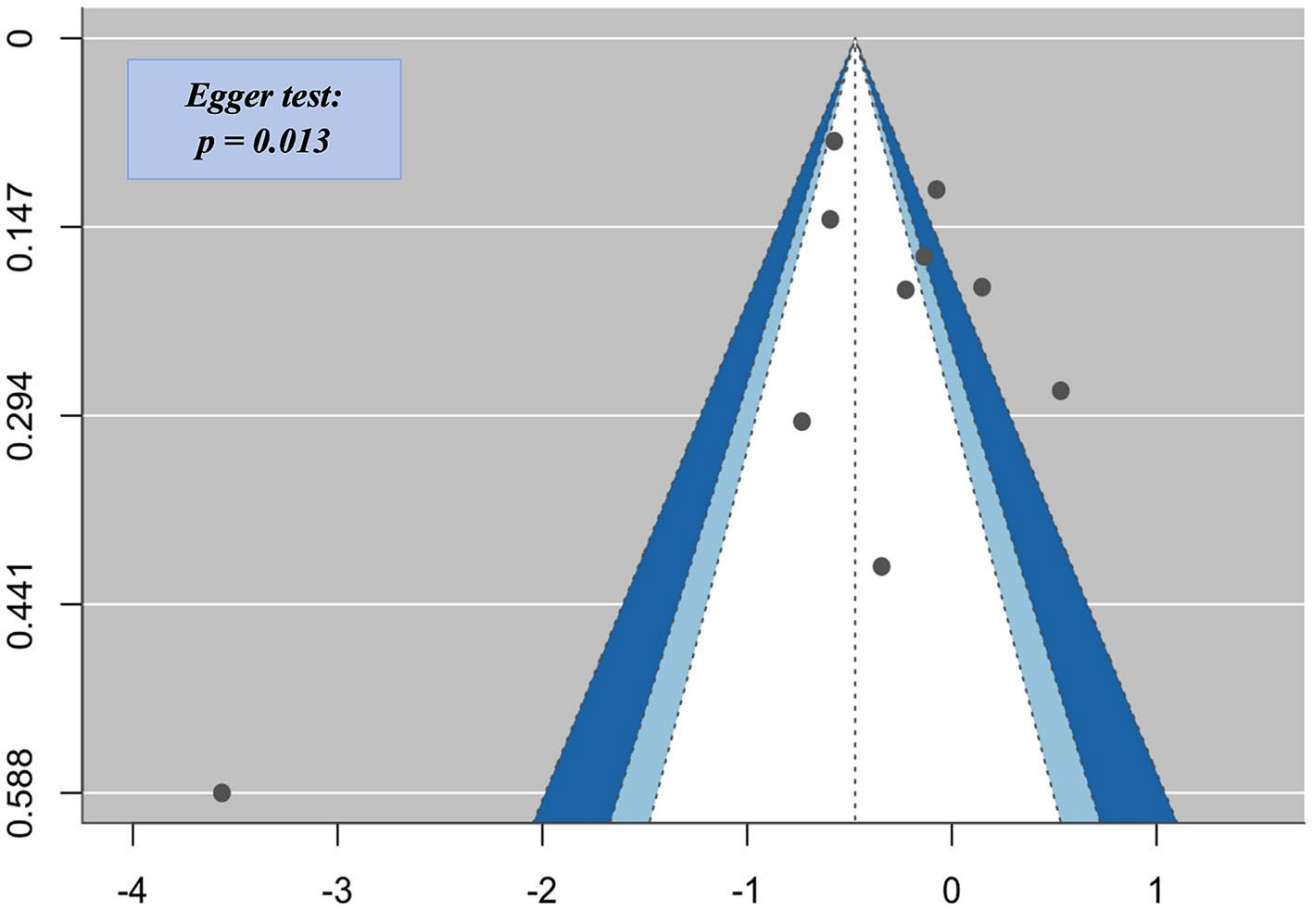
Funnel plot for aggression outcomes showing evidence of publication bias.

Regarding publication bias, funnel plots and Egger’s regression test indicated evidence of asymmetry for aggression (z = −2.49, *p* = 0.013), suggesting potential publication bias (Figure 4). Publication bias was not assessed for hostility and anger due to the limited number of studies.

### Sensitivity analysis

For the aggression outcome, the leave-one-out sensitivity analysis demonstrated that the pooled effect estimates remained stable when any single study was omitted (see Figure 1A). The standardized mean differences (SMDs) ranged from 0.23 to 0.57, and all corresponding 95% confidence intervals continued to cross zero, indicating that the direction and statistical significance of the overall effect were not materially altered. The pooled estimate after exclusions was consistent with the main analysis (SMD = 0.46, 95% CI − 0.24 to 1.16). Regarding heterogeneity, I^2^ values fluctuated between 78.3 and 87.6%, while Tau^2^ (0.10–0.84) and Tau (0.31–0.92) also remained relatively stable, suggesting that no individual study disproportionately influenced the degree of heterogeneity. Taken together, these findings confirm the robustness of the meta-analysis results, with no single study exerting a decisive impact on the overall conclusions.

For the hostility outcome, the leave-one-out sensitivity analysis revealed that the pooled effect estimates remained largely consistent after sequentially omitting individual studies (see Figure 1B). The standardized mean differences (SMDs) ranged from 0.23 to 0.31, and the 95% confidence intervals mostly remained statistically significant, except when excluding Shachar et al. (2016) where the CI marginally crossed zero. The pooled estimate (SMD = 0.29, 95% CI 0.13 to 0.45) was highly consistent with the main analysis. Heterogeneity was minimal across all analyses, with I^2^ values ranging from 0 to 9% and Tau^2^ values close to zero, indicating negligible between-study variability. These findings suggest that the observed beneficial effect of sports interventions on hostility was robust and not driven by any single study.

For anger, the leave-one-out analysis showed that the pooled effect sizes ranged from 0.24 to 0.46, with the direction of effect remaining consistent across all iterations. In most cases, the 95% confidence intervals crossed the null value, and heterogeneity remained substantial (I^2^ ≈ 78–82%). Notably, the exclusion of Reynes’s study (Reynes and Lorant, 2002) resulted in a statistically significant pooled effect (SMD = 0.46, 95% CI 0.31 to 0.62) and eliminated heterogeneity (I^2^ = 0%), suggesting that this study may have been a major source of between-study variability. Nevertheless, because *post hoc* exclusion without predefined criteria can introduce bias, Reynes’s study (Reynes and Lorant, 2002) was retained in the primary analysis, and the main findings should be interpreted in light of this heterogeneity (Figure 1C).

## Main effect results

### Aggression

**Figure 5:**
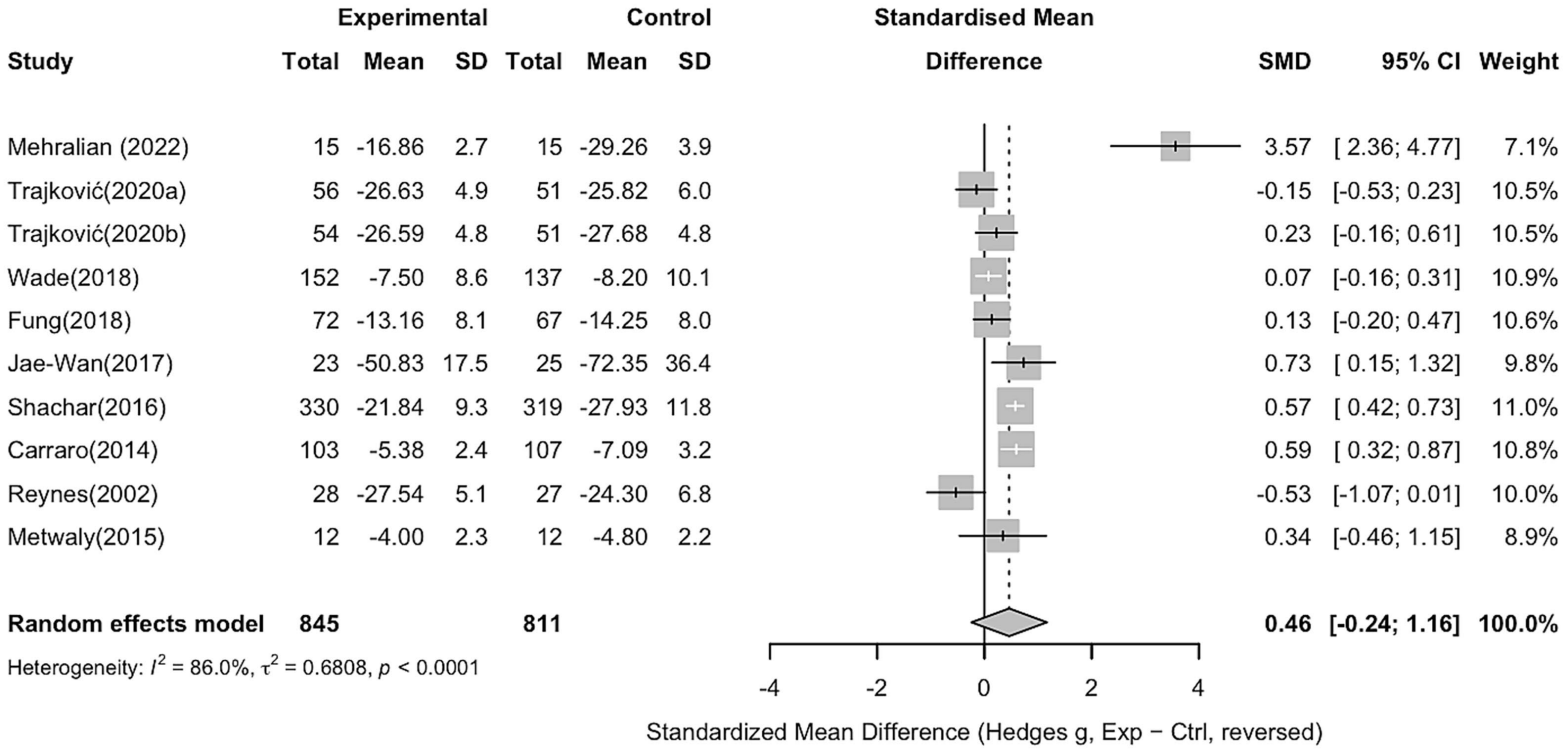
Forest plot showing the effect of sports interventions on aggression compared to controls (random effects model).

For aggression, 10 studies with 845 participants in the intervention group and 811 in the control group were included. The pooled analysis showed no significant overall effect of sports interventions compared to controls (Hedges’ g = 0.46, 95% CI − 0.24 to 1.16), with considerable heterogeneity (I^2^ = 86%). Several individual studies reported significant reductions in aggression, while others showed non-significant or opposite effects, with specific results shown in Figure 5.

**Figure 6:**
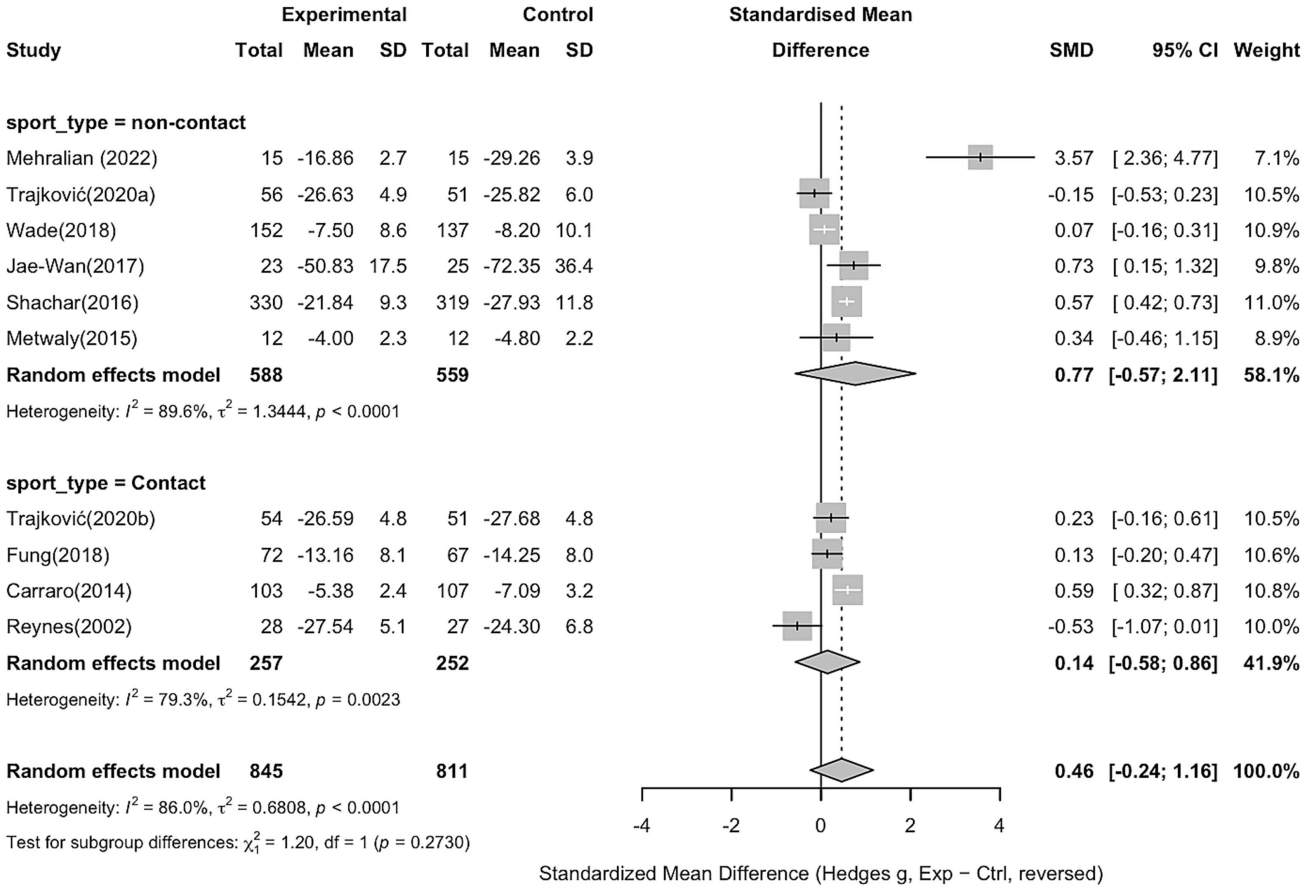
Subgroup analysis of aggression based on sport type (non-contact vs. contact sports).

To explore potential sources of heterogeneity, a subgroup analysis was conducted based on sport type (non-contact vs. contact sports). Non-contact sports showed a non-significant effect compared to controls (Hedges’ g = 0.77, 95% CI − 0.57 to 2.11). Similarly, contact sports also did not demonstrate a significant effect (Hedges’ g = 0.14, 95% CI − 0.58 to 0.86), with the detailed results shown in Figure 6.

### Hostility

**Figure 7:**
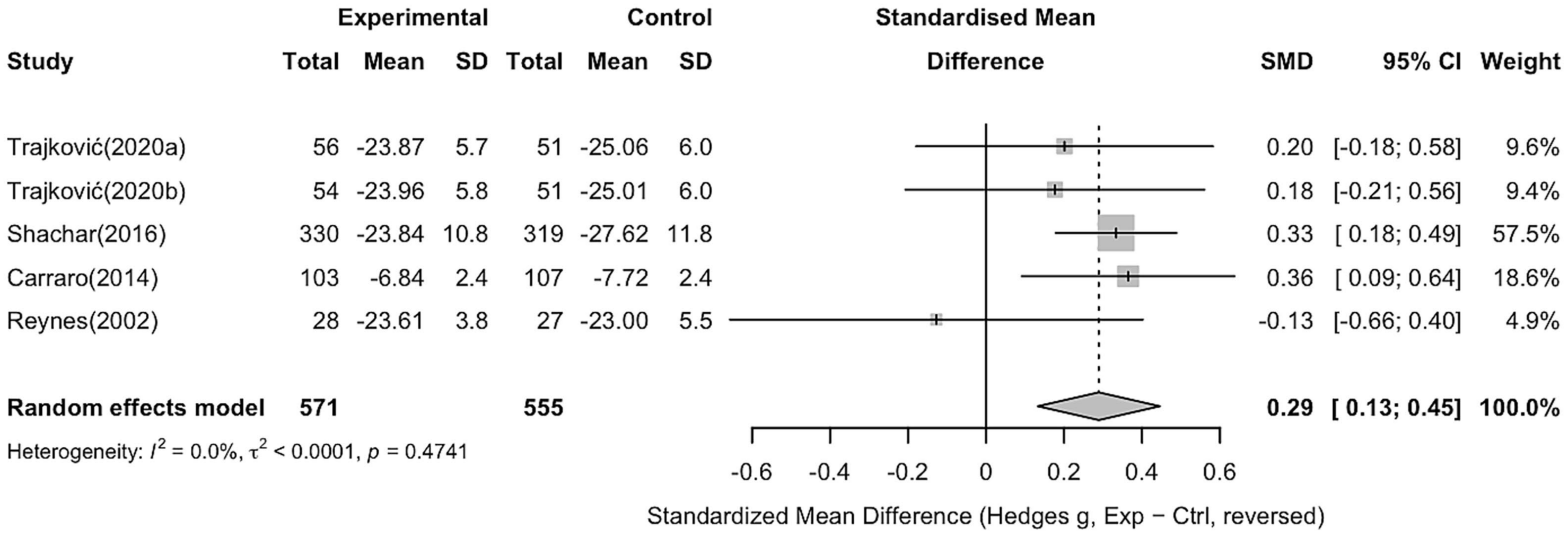
Forest plot showing the effect of sports interventions on hostility compared to controls (random effects model).

For hostility, five studies with 571 participants in the intervention group and 555 in the control group were included. The pooled analysis showed a significant effect of sports interventions compared to controls (Hedges’ g = 0.29, 95% CI 0.13 to 0.45), indicating a reduction in hostility. No heterogeneity was observed across the studies (I^2^ = 0.0%, *p* = 0.47), suggesting consistent effects among the included trials (see Figure 7).

### Anger

**Figure 8:**
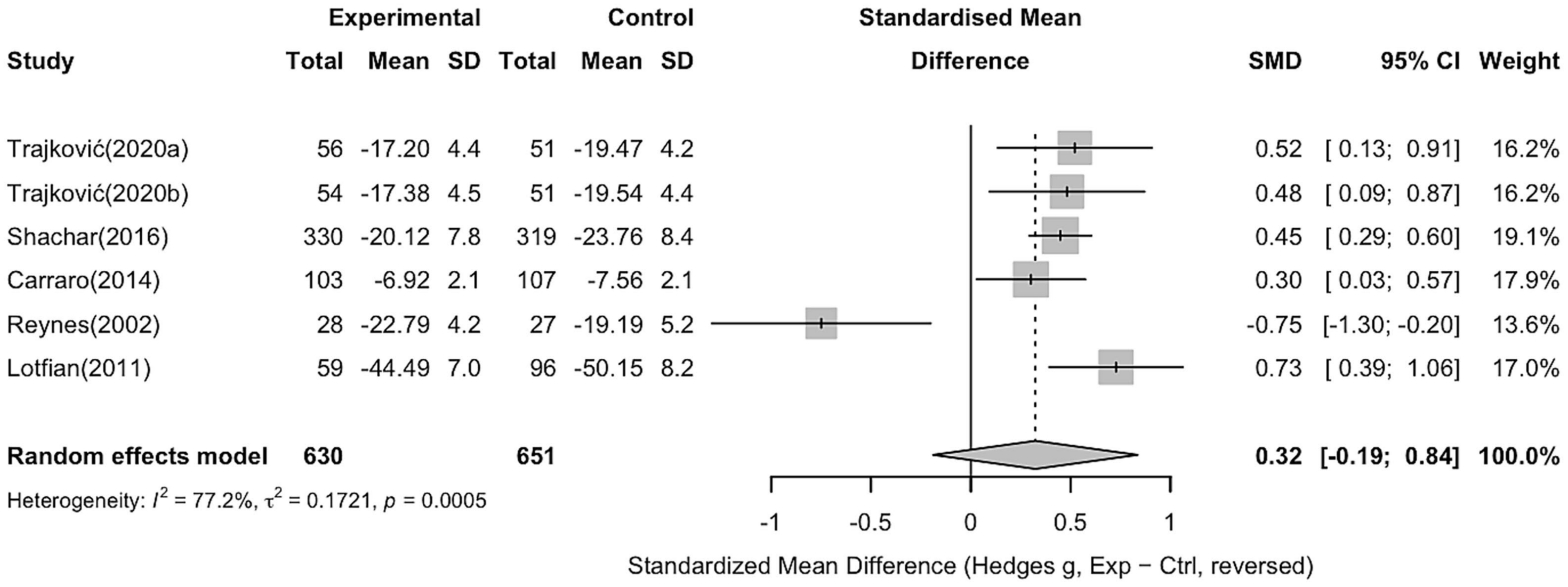
Forest plot showing the effect of sports interventions on anger compared to controls (random effects model).

For anger, six studies with 630 participants in the intervention group and 651 in the control group were included. The pooled analysis showed no significant effect of sports interventions compared to controls (Hedges’ g = 0.32, 95% CI − 0.19 to 0.84). Substantial heterogeneity was observed across the studies (I^2^ = 77.2%, *p* = 0.0005), indicating considerable variability in intervention effects (see Figure 8).

**Figure 9:**
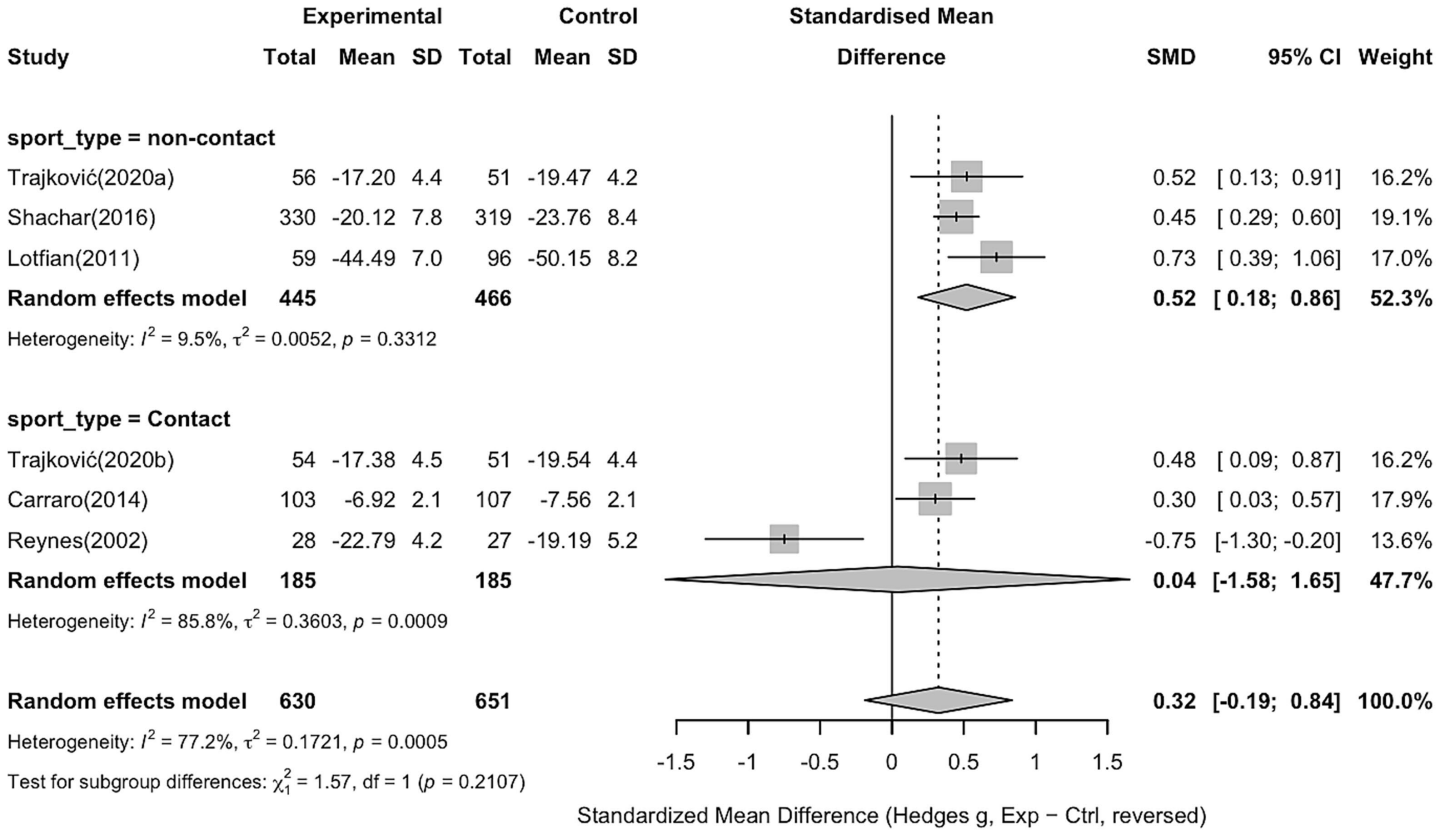
Subgroup analysis of anger based on sport type (non-contact vs. contact sports).

To explore potential sources of heterogeneity, a subgroup analysis was conducted based on sport type (non-contact vs. contact sports). Non-contact sports showed a significant effect compared to controls (Hedges’ g = 0.52, 95% CI 0.18 to 0.86). Contact sports showed a non-significant effect (Hedges’ g = 0.04, 95% CI − 1.58 to 1.65), with the detailed results shown in Figure 9.

## Discussion

This systematic review and meta-analysis examined the effects of sports interventions on aggression-related outcomes in adolescents. Three key outcomes—aggression, hostility, and anger—were analyzed. The results indicated a significant reduction in hostility but no significant effects on aggression or anger. Subgroup analyses by sport type revealed differing effects for aggression and anger. For aggression, non-contact sports showed a non-significant effect compared to controls, while contact sports also did not demonstrate a significant effect, suggesting a limited impact on aggression-related behaviors for both types of sports. Furthermore, for anger, non-contact sports showed a significant effect compared to controls, while contact sports showed a non-significant effect. These findings suggest that while sports interventions may help mitigate hostility, their overall impact on aggression-related behaviors, including aggression and anger, remains inconclusive. Furthermore, considerable heterogeneity across studies, particularly regarding aggression and anger outcomes, underscores the complexity of this relationship.

### Aggression

Our findings on aggression are inconsistent with existing literature, where some studies report a reduction in aggression following sports participation (Yang et al., 2023). However, the pooled analysis revealed no significant overall effect of sports interventions on aggression, with a high degree of heterogeneity across the studies. Subgroup analyses by sport type revealed no significant effect for either non-contact or contact sports, suggesting that sport type may not be a major determinant of aggression reduction in this context, which is inconsistent with some existing studies that have found sport type to influence aggression outcomes (Pels and Kleinert, 2016).

These findings indicate that the efficacy of sports interventions in reducing aggression is inconsistent, which may be partly due to differences in intervention duration and frequency. For example, when the intervention duration was equal to or more than six months, sports interventions were not associated with lower aggression (Yang et al., 2023). Similarly, Stansfield (2017) reported that higher levels of sports participation were linked to increased involvement in violent behaviors, while.

another study (2019) found that students with high exercise frequency exhibited greater aggression than those with lower activity levels. These findings suggest that longer or more intensive participation in sports does not necessarily reduce aggression and, in some contexts, may even reinforce competitive or confrontational tendencies.

### Hostility

In contrast, hostility was significantly reduced following sports interventions, with no heterogeneity observed. This suggests that sports interventions may specifically target the cognitive and attitudinal components of aggression, such as hostility, which are closely related to social and emotional regulation (Xu et al., 2025).

One possible explanation is that hostility reflects internalized attitudes rather than overt behaviors (Nelson and Perry, 2015), making it more susceptible to changes in emotional regulation and social cognition fostered by sport participation. Engaging in sports can enhance self-awareness, empathy, and perspective-taking, thereby strengthening socio-emotional competencies such as self-regulation, cooperation, and respect for rules (Filges et al., 2024). Through these processes, individuals may reinterpret provocation or competition in less adversarial ways, leading to reductions in hostile attitudes. Collectively, these mechanisms may explain why hostility appears more responsive to sports interventions than other dimensions of aggression.

### Anger

We found that non-contact sports showed a significant reduction in anger compared to the control group, while contact sports did not show significant effects. This suggests that non-contact sports may be more effective in alleviating anger, possibly due to their lower levels of physical contact and competition intensity. This result is consistent with previous studies (Pels and Kleinert, 2016).

Drawing on data from 141 athletes representing a range of sport types, Sofia and Cruz (2017) demonstrated that aggression levels were higher among athletes involved in high-contact sports than among those in low-contact disciplines. They argued that this discrepancy could be attributed to the role of self-control as a key regulatory mechanism of aggression within competitive settings (Sofia and Cruz, 2015; Sofia and Cruz, 2016). High-contact sports are often characterized by heightened competitiveness and increased impulsivity, which may increase the likelihood of anger arousal. In contrast, participants in non-contact sports are less exposed to physical confrontation and may develop better emotional regulation and self-control. Furthermore, Pels and Kleinert (2016) compared rowing and combat exercise and reached a similar conclusion: the non-contact sport of rowing was more effective in reducing aggression than high-contact combat activities. Together, these findings suggest that the intensity of physical contact and competition may act as key moderating factors influencing anger outcomes, with non-contact sports providing a more favorable environment for emotional regulation and self-control.

### Implications of the findings

The present findings offer practical guidance for educators, coaches, and parents involved in youth development. Incorporating non-contact or cooperative sports into daily training or recreational activities may be particularly beneficial for managing aggression and anger among adolescents. For youth intervention programs, sports can serve as an effective behavioral and emotional regulation tool. Programs that emphasize enjoyment, teamwork, and positive reinforcement, rather than competition and confrontation, may help reduce aggression-related tendencies and promote healthier emotional development.

### Limitations and future directions

First, the studies included in this meta-analysis varied in terms of intervention duration, frequency, and sport type, which may have contributed to the observed heterogeneity. Although additional subgroup analyses were performed to explore potential sources of heterogeneity (e.g., by sport type), the variability remained high. Due to the limited number of studies and inconsistent reporting of potential moderators such as intervention duration, frequency, or intervention type, further subgroup or meta-regression analyses were not feasible. In addition, most studies relied on self-report measures of aggression-related outcomes, which are susceptible to social desirability bias and may not fully capture the true extent of aggression. Moreover, cultural differences and the social environments of adolescents may influence how sports interventions affect aggression, which could limit the generalizability of the findings.

Future research should address these limitations by employing more rigorous experimental designs and investigating the long-term effects of sports interventions on aggression. At the same time, it should explore the underlying mechanisms through which sports influence aggression, such as changes in self-control, social skills, and emotional regulation.

## Conclusion

This systematic review and meta-analysis found that sports interventions may reduce hostility but have no significant impact on aggression and anger in adolescents. Non-contact sports showed a significant effect in reducing anger, but no significant effects were observed for aggression. Meanwhile, no significant effects were found for aggression or anger in contact sports, suggesting that while sports interventions could help mitigate hostility, their effectiveness in addressing aggression and anger requires further investigation. Future research should adopt longitudinal and rigorously controlled designs to verify the long-term effects of sports interventions and explore the psychological mechanisms, such as emotional regulation, self-control, and social skills, through which sports may influence aggression-related outcomes.

## Data Availability

The curated extraction dataset and meta-analysis code are available from the corresponding author upon reasonable request.
