# Supplementary material for: Impact of sports interventions on aggressive behavior among adolescents: a systematic review and meta-analysis
Source: Front Psychol. 2025 Nov 6;16:1697324. doi: 10.3389/fpsyg.2025.1697324 (PMC12634051; doi:10.3389/fpsyg.2025.1697324)
Supplement: Supplementary file 1 [file Table_1.docx]

**Appendix 1：search strategy**

| **Database** | **Retrieval strategy** |
| --- | --- |
| **Cochranne** | #1 (exercise:ti,ab,kw OR sport*:ti,ab,kw OR (physical NEXT activit*):ti,ab,kw  OR (physical NEXT education):ti,ab,kw OR (fitness NEXT training):ti,ab,kw OR (motor NEXT activit*):ti,ab,kw)  #2 (adolescent*:ti,ab,kw OR teen*:ti,ab,kw OR youth:ti,ab,kw  OR (young NEXT people):ti,ab,kw OR (high NEXT school NEXT student*):ti,ab,kw  OR (middle NEXT school NEXT student*):ti,ab,kw OR juvenile*:ti,ab,kw)  #3 (aggression:ti,ab,kw OR (aggressive NEXT behavior):ti,ab,kw OR (aggressive NEXT behaviour):ti,ab,kw  OR hostility:ti,ab,kw OR (hostile NEXT behavior):ti,ab,kw OR (hostile NEXT behaviour):ti,ab,kw  OR bullying:ti,ab,kw OR fighting:ti,ab,kw)  #4 #1 AND #2 AND #3 |
| **Embase** | ( 'exercise'/exp OR 'sport'/exp OR 'physical education'/exp OR 'physical activity'/exp OR exercise:ti,ab OR sport*:ti,ab OR "physical activit*":ti,ab OR "physical education":ti,ab OR "fitness training":ti,ab OR "motor activit*":ti,ab ) AND ( 'adolescent'/exp OR adolescent*:ti,ab OR teen*:ti,ab OR youth:ti,ab OR "young people":ti,ab OR "high school student*":ti,ab OR "middle school student*":ti,ab OR juvenile*:ti,ab ) AND ( 'aggression'/exp OR 'bullying'/exp OR aggression:ti,ab OR "aggressive behavio?r":ti,ab OR hostility:ti,ab OR "hostile behavio?r":ti,ab OR bullying:ti,ab OR fighting:ti,ab ) |
| **Web of Science** | TS=(("exercise" OR "sport*" OR "physical activit*" OR "physical education"  OR "fitness training" OR "motor activit*")  AND ("adolescent*" OR "teen*" OR "youth" OR "young people"  OR "high school student*" OR "middle school student*" OR "juvenile*")  AND ("aggression" OR "aggressive behavio*r" OR "hostility" OR "hostile behavio*r"  OR "bullying" OR "fighting")) |
| **PubMed** | ( "Exercise"[Mesh] OR "Sports"[Mesh] OR "Physical Education and Training"[Mesh] OR "Motor Activity"[Mesh] OR exercise[Title/Abstract] OR sport*[Title/Abstract] OR "physical activit*"[Title/Abstract] OR "physical education"[Title/Abstract] OR "fitness training"[Title/Abstract] OR "motor activit*"[Title/Abstract] ) AND ( "Adolescent"[Mesh] OR adolescent*[Title/Abstract] OR teen*[Title/Abstract] OR youth[Title/Abstract] OR "young people"[Title/Abstract] OR "high school student*"[Title/Abstract] OR "middle school student*"[Title/Abstract] OR juvenile*[Title/Abstract] ) AND ( "Aggression"[Mesh] OR "Bullying"[Mesh] OR aggression[Title/Abstract] OR "aggressive behavio*r"[Title/Abstract] OR hostility[Title/Abstract] OR "hostile behavio*r"[Title/Abstract] OR bullying[Title/Abstract] OR fighting[Title/Abstract] ) |
| **Sportdiscus** | (TI(exercise OR sport* OR "physical activit*" OR "physical education" OR "fitness training" OR "motor activit*") OR AB(exercise OR sport* OR "physical activit*" OR "physical education" OR "fitness training" OR "motor activit*") OR SU(exercise OR sport* OR "physical activity" OR "physical education" OR "fitness training" OR "motor activity"))AND(TI(adolescent* OR teen* OR youth OR "young people" OR "high school student*" OR "middle school student*" OR juvenile*) OR AB(adolescent* OR teen* OR youth OR "young people" OR "high school student*" OR "middle school student*" OR juvenile*) OR SU(adolescent* OR teen* OR youth OR "young people" OR "high school students" OR "middle school students" OR juveniles))AND(TI(aggression OR "aggressive behavior" OR "aggressive behaviour" OR hostility OR "hostile behavior" OR "hostile behaviour" OR bullying OR fighting) OR AB(aggression OR "aggressive behavior" OR "aggressive behaviour" OR hostility OR "hostile behavior" OR "hostile behaviour" OR bullying OR fighting) OR SU(aggression OR "aggressive behavior" OR "aggressive behaviour" OR hostility OR "hostile behavior" OR "hostile behaviour" OR bullying OR fighting)) |
